# Adriamycin‐loaded exosome with anti‐CD20 aptamers selectively suppresses human CD20+ melanoma stem cells

**DOI:** 10.1111/srt.13259

**Published:** 2022-12-23

**Authors:** Hairong Chen, Yuxia Jiang, Xia Li

**Affiliations:** ^1^ Department of Dermatology The Affiliated Qingdao Municipal Hospital of Qingdao University Qingdao Shandong China; ^2^ Department of Clinical Laboratory The Affiliated Qingdao Municipal Hospital of Qingdao University Qingdao Shandong China; ^3^ Department of Dermatology The West District of Qingdao Municipal Hospital Group (Qingdao Ninth People's Hospital) Qingdao Shandong China

**Keywords:** Adriamycin, aptamer, cancer stem cell, exosome, melanoma

## Abstract

**Background:**

Targeting CD20+ melanoma cancer stem cells (CSCs) subset is essential for treating melanoma. Anti‐CD20 aptamer‐modified exosomes (ACEXO) loaded with Adriamycin could be a therapeutic strategy for targeting CSCs.

**Materials and methods:**

Exosomes loaded with Adriamycin were modified with anti‐CD20 aptamer and characterized by size and molecular markers using transmission electron microscope and dynamic light scattering. The uptake of ACEXO into CD20+ cells was checked, and its cytotoxicities in CD20+ melanoma cells, HEK 293T, and 3T3 cells were evaluated. At the same time, the in vivo distribution of ACEXO in the tumor‐bearing mice model was determined.

**Results:**

The particle size of the exosome is about 80–100 nm. Western blot analysis showed that they expressed the characteristic exosome markers: CD9 and CD63. Quantitative analysis of the mean fluorescence intensity after 4 h incubation showed that ACEXO significantly improved Adriamycin uptake. Notably, the ACEXO killed only CD20+ melanoma cells. In addition, they exhibited good biocompatibility with both 293T and 3T3 cells at all doses. After intravenous injection, exosome distribution data showed that ACEXO's accumulation in the tumor is higher than anti‐CD20‐modified exosomes (AEXO)’s at all time points, and the accumulation increased as time prolonged. Addition of ACEXO reduces the number of tumorspheres in A375 or WM266‐4 cells compared to untreated controls or AEXO‐treated group. More important, while treating melanoma tumor‐bearing mice, ACEXO‐treated group showed the lowest tumor weight without body weight loss.

**Conclusion:**

ACEXO loaded with Adriamycin could suppress tumor cell growth in vitro and in vivo, probably by targeting CD20+ melanoma CSCs.

## 1 INTRODUCTION

Melanoma develops from melanocytes. It is an aggressive skin cancer resistant to many chemotherapy drugs.^1^ Although melanoma is a rare type of skin cancer (1% of skin cancers), it is the deadliest type of skin cancer, and the fact that the majority of skin cancer deaths are caused by melanoma. Over the past 30 years, the incidence of melanoma in the United States and worldwide has been rising.^2^ Therefore, it is imperative to develop effective treatments to treat melanoma. Cancer stem cells (CSCs) are key to tumor initiation and recurrence in cancer.^3^, ^4^ Previous studies have identified melanoma CSCs characterized by the marker CD20.^5^ In melanoma tissues and cell lines, CD20+ melanoma CSCs can self‐renew, differentiate, and cause tumors. It is important to note that eliminating CD20+ melanoma cells can permanently eradicate melanoma, while eliminating other melanoma subsets cannot. Notably, the anti‐CD20 antibody Rituximab shows potential therapeutic value in patients with stage 4 metastatic melanoma.^6^ Therefore, the CD20+ melanoma CSCs subset is essential for melanoma growth. Thus, selective clearance of CD20+ melanoma CSCs is an effective treatment for melanoma eradication. As a traditional chemotherapy drug for treating many cancers, Adriamycin (also known as Doxorubicin) is limited in treating melanoma CSCs due to its poor solubility and significant toxic side effects.^7^ However, a targeted therapeutic strategy using Adriamycin would benefit patients from by reducing its toxicity.

Exosomes are used for targeted drug delivery.^8^ They are extracellular membrane vesicles with a diameter of 40–200 nm, and almost all cell types can secrete exosomes and are stable in body fluids. Exosomes as natural carriers can be used as drug delivery carriers, with various advantages over existing drug delivery systems. On the one hand, properly sized exosomes allow them to escape phagocytosis. On the other hand, exosomes can come from the patient's cells, minimizing cytotoxicity and immunogenicity.

Exosomes with ligands can significantly improve targeting efficiency. Antibodies are commonly used as ligand target nanoparticles, but their strong immunogenicity and high molecular weight greatly limit their application. Aptamers are single‐stranded nucleic acids that selectively bind to the molecule of interest.^9^ They have the advantages of low molecular weight and non‐immunogenicity and are readily available. ACDA is an anti‐CD20 DNA aptamer with a stronger binding affinity with CD20 than the Fab antibody fragment of Rituximab.^5^ It can mediate specificity and efficient nanoparticle delivery to CD20+ melanoma CSCs. To target CD20+ melanoma CSCs, we designed anti‐CD20 aptamer‐modified exosomes (ACEXO) loaded with Adriamycin.

## 2 METHODS AND MATERIALS

### 2.1 Preparation of exosomes

Exosomes with iRGD cyclic peptide modified on the exosome membrane surface were prepared. The peptide sequence is CRGDKGPDC, and DSPE‐PEG2000‐mal was incorporated into EXO by post‐insertion method. To conjugate CD20 aptamer to exosomes, exosomes (2 ml) were treated with 0.5 ml of CD20 aptamer (1 mg/ml) for 2 h with stirring. Free aptamers are subsequently removed by dialysis. The modified exosomes were further co‐incubated with Adriamycin for half an hour to obtain the specific exosomes. Unless otherwise specified, cell experiments were performed at a membrane protein concentration of 200 μg/ml. CD20 aptamer (sequence: 5′‐SH‐CTCCTCTGACTGTAACCACGCCGTATGTCCGAAATACGGAGAACAGCACTCATATGCAAGCCATACGCGGAGGTGCACGCGCATAGGTAGTCCAGAAGCC‐3′) was fabricated by Ruibo Co, Ltd (Guangzhou, China).

### 2.2 Exosome characterization

Two methods are used for determine the size of exosomes, including the transmission electron microscope (TEM) and dynamic light scattering (DLS) methods, following routine procedures.

### 2.3 Melanoma cell lines

WM266‐4 and A375 are human melanoma cell lines, purchased from American Type Culture Collection (ATCC, Manassas, VA). The cells were verified by STR analysis before use. Cells were maintained in DMEM with fetal bovine serum (FBS, 10%, Gibco), penicillin (100 U/ml), streptomycin (100 μg/ml), and hydroxyethyl piperazine ethanesulfonic acid buffer (25 mmol/L), in a humidified atmosphere of 5% CO_2_ at 37°C.

### 2.4 Western blot

Protein concentrations were detected before blotting, determined by the BCA method. The protein sample is denatured by heating at 95°C for 5 min. An amount of 20 μg protein was loaded into each lane on a 10% SDS‐PAGE gel. After transferring the protein to a PVDF membrane, the sub‐membrane blocks with 5% skim milk for 1 h at room temperature, followed by incubation with primary antibodies, including CD9, CD63, and GAPDH (all from cell signaling) overnight in a blocking buffer at 4°C. The membrane is then washed three times with TBST for 5 min each incubated with secondary antibody for 1 h in blocking buffer at room temperature. After washing, the membrane is treated with an ECL substrate (abs921, Absin) to acquire images. Images were obtained by using darkroom development techniques for chemiluminescence. GAPDH is used as an internal reference control.

### 2.5 Cell proliferation assay

For the cell proliferation test, cells were seeded in 96‐well plates (1 × 10^4^ cells/well) for 12 h. Various concentrations of exosomes were added to the cells, and cells were incubated for 72 h. Then, cell viability was measured using MTT assay (Sigma, St. Louis, MO).

### 2.6 Animal studies

Severe combined immunodeficient (SCID) mice (∼20 g, 6 weeks) were bought from the Cyagen Biosciences Inc, Suzhou, China. All procedures were approved by the Affiliated Qingdao Municipal Hospital of Qingdao University and performed in accordance with the guidelines. The in vivo tumorigenicity assay was performed in SCID mice as described later. Briefly, melanoma cells (2 × 10^5^) were subcutaneously implanted into SCID mice. The tumor formation of the cells was monitored for 60 days.

### 2.7 Statistical analysis

Statistical analysis was performed using Student's *t* test, one‐ or two‐way analysis of variance (ANOVA) test followed by a post hoc test, using GraphPad software. Significant difference was determined when *p* value is less than 0.05.

## 3 RESULTS

### 3.1 Characterization of ACEXO

**FIGURE 1:**
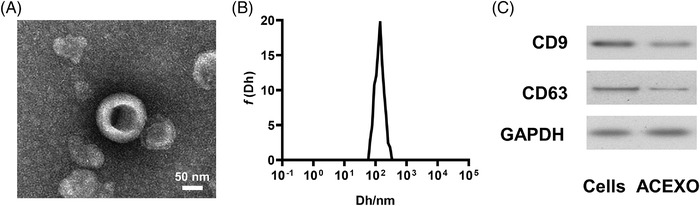
Characterization of ACEXO: (A) representative transmission electron microscopy (TEM) image of ACEXO; (B) size distributions of ACEXO; and (C) Western blot analysis of CD9 and CD63 from HEK 293T cells and exosomes

First, the isolated exosomes were identified from the features by using TEM and DLS. Judging from the results of TEM, the particle size of the exosome is about 80–120 nm (Figure 1A). In the results of DLS, most of the particles are around 100 nm (Figure 1B), which indicates that the particles are among the size of exosomes (40–200 nm). In addition, Western blot analysis showed that the obtained pellets expressed the characteristic exosome markers CD9 and CD63 (Figure 1C). These results indicated that exosomes were successfully prepared. Then the exosomes were utilized for modification with an anti‐CD20 aptamer for further experiments.

### 3.2 Uptake of ACEXO in vitro and in vivo

**FIGURE 2:**
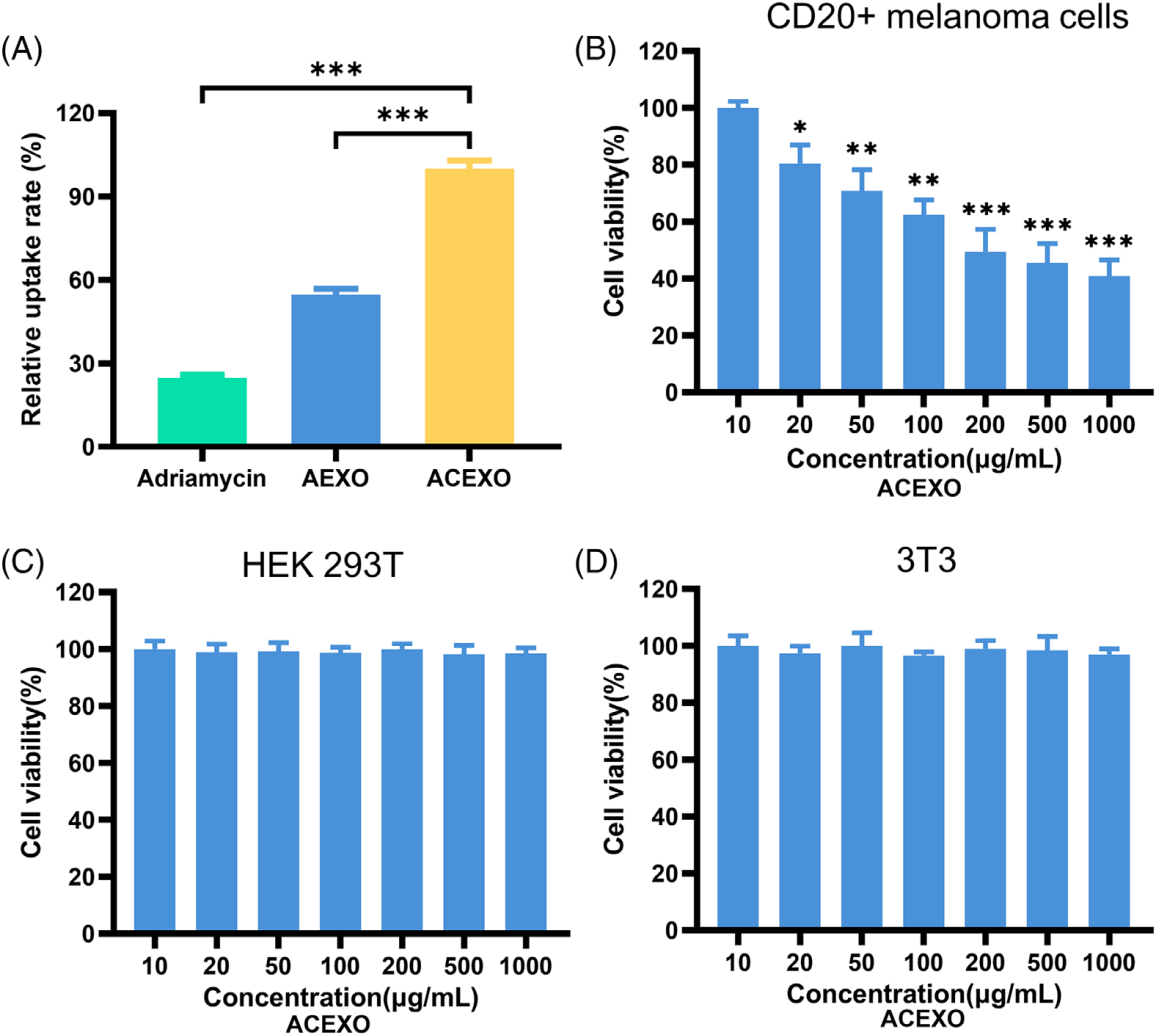
Quantification for uptake of ACEXO. (A) In vitro cellular uptake of exosomes in CD20+ melanoma cells. Cytotoxicity of ACEXO in CD20+ melanoma cells (B), HEK 293T (C), and 3T3 cells (D). Data were represented as mean ± SD, *n* = 6. “***” Indicating *p* < 0.001, “**” indicating *p* < 0.01, and “*” indicating *p* < 0.05. Statistics were conducted using one‐way analysis of variance (ANOVA) followed by a Tukey's post hoc test

ACEXO's ability to deliver Adriamycin to target cells is critical to therapeutic efficacy. Here, Adriamycin (Adriamycin excitation wavelength 469 nm, the emission is 593 nm) is used as a fluorescence index to monitor the internalization of ACEXO into CD20+ cells. Quantitative analysis of the mean fluorescence intensity of flow cytometry assays after 4 h incubation confirmed that ACEXO significantly improved Adriamycin uptake (Figure 2A). In the drug delivery process, cytotoxicity limits its use. We used the MTT method to test the cytotoxicity of ACEXO in CD20+ melanoma cells, HEK 293T, and 3T3 cells. As shown in Figure 2B–D, ACEXO killed only CD20+ melanoma cells and exhibited good biocompatibility with 293T and 3T3 cells at all doses. These results confirmed the high biocompatibility of exosomes as drug delivery carriers.

**FIGURE 3:**
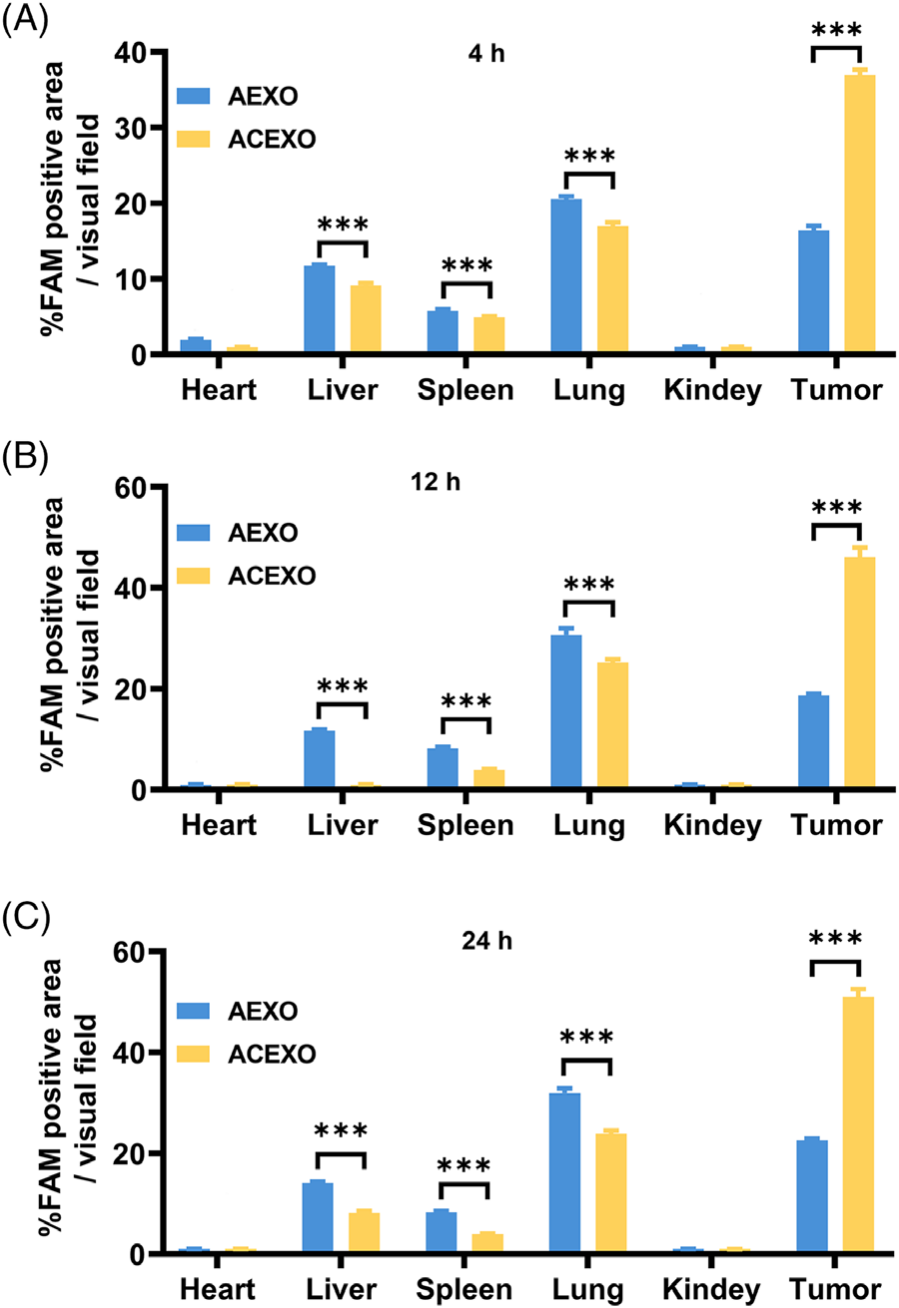
In vivo distribution of ACEXO after intravenous injection. Quantification analysis of ACEXO in tissues, including heart, liver, spleen, lung, kidney, and tumor in mice. At different time points, the positive areas were calculated, including 4 h (A), 12 h (B), and 24 h (C) after injection. Data were represented as mean ± SD, *n* = 6. “***” Indicating *p* < 0.001. Statistics were conducted using Student's *t* test

Next, we texted the in vivo distribution of ACEXO. After intravenous injection, exosome distribution data showed that ACEXO's accumulation in the tumor was higher than AEXO's at all time points (Figure 3A–C), especially at 24 h (Figure 3C), supporting the conclusion that CD20 aptamer‐modified exosomes have a specific tendency toward tumors. Specifically, the fluorescence intensity of the ACEXO treatment group was higher than that of 4 h at 12 h. By 24 h, the fluorescence signal intensity of tumor tissue was further increased, indicating that the tumor tissue targeting and adhesion ability were excellent. In contrast, although continuously enhanced, the fluorescence signal in the AEXO treatment group showed a significant difference in intensity from ACEXO. Taking together, ACEXO showed a character with low cytotoxicity for non‐tumor cells and a higher affinity for tumor cells.

### 3.3 Effect of ACEXO on CSCs

**FIGURE 4:**
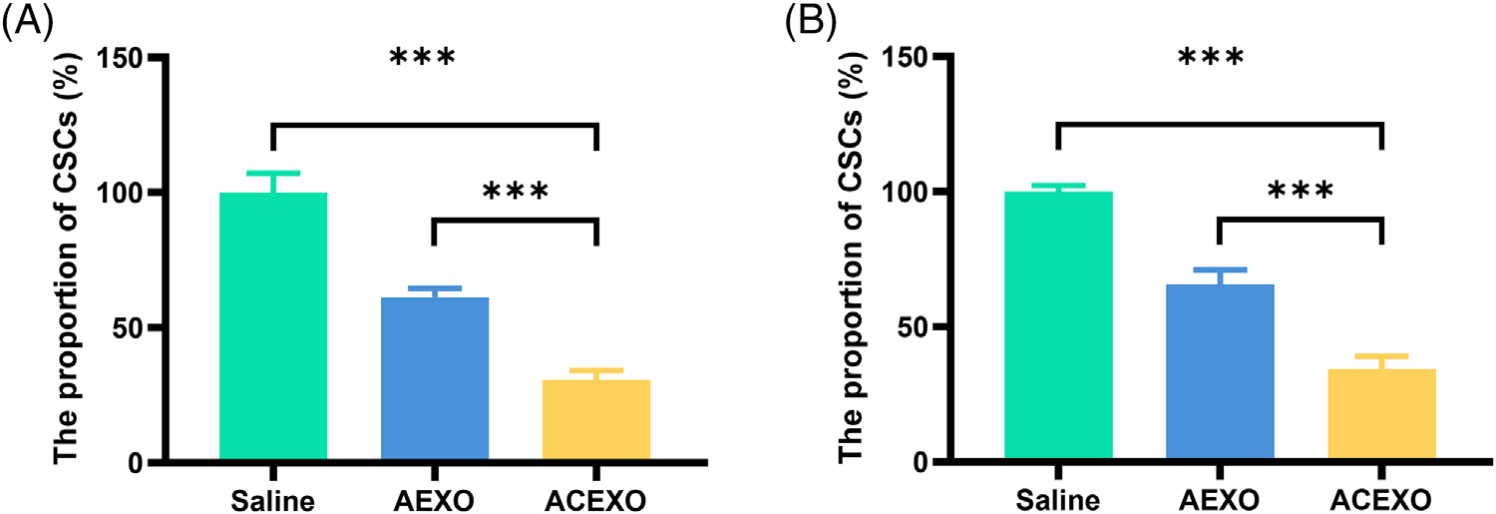
The effect of drugs on the proportion of cancer stem cells (CSCs). In (A) A375 and (B) WM266‐4 cells, as reflected by the percentage of tumor sphere formation. All results are compared by one‐way analysis of variance (ANOVA) with the Newman–Keuls post‐test; “***” *p* < 0.001. Data are expressed as the mean SD (*n* = 6). Statistics were conducted using one‐way analysis of variance (ANOVA) followed by a Tukey's post hoc test

Targeting CSCs will maximize tumor suppression, and we then explored whether ACEXO can target CSCs. The percentage of tumor ball formation is thought to be related to the ratio of CSCs, and the effect of ACEXO on CSCs is tested by detecting tumor ball formation in A375 and WM266‐4 cells. Figure 4A shows that ACEXO reduces the number of A375 tumor balls compared to untreated controls, resulting in fewer tumor balls forming compared to AEXO‐treated group. For WM266‐4 cells, we obtained similar results (Figure 4B). ACEXO reduces the number of tumor balls compared to untreated controls and results in fewer tumor balls forming compared to AEXO‐treated group. These results suggested that ACEXO could better target CSCs.

### 3.4 Effect of ACEXO in mouse tumor model

**FIGURE 5:**
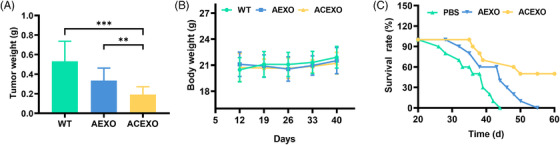
Effect of exosomes in tumor‐bearing mouse model: (A) tumor weight in each group after treatment; (B) body weight, and (C) survival rates of the three experimental groups of tumor‐bearing mice during the 12‐day treatment. Data were represented as mean ± SD (*n* = 6). “***” Indicating *p* < 0.001, “**” indicating *p* < 0.01. Statistics were conducted using one‐way analysis of variance (ANOVA) followed by a Tukey's post hoc test for panel a, two‐way ANOVA followed by a Bonferroni post hoc test for panels B and C

To further test the tumor suppressor effect, we detected the effect of ACEXO through a tumor model in mice. At the end of the experiment, mice were sacrificed, and tumor tissues were collected for analysis. There was a significant difference in average tumor weight between the three groups, ACEXO‐treated group showed the lowest tumor weight (Figure 5A). However, ACEXO‐treated mice did not have significant differences in weight loss between groups (Figure 5B), suggesting that ACEXO may inhibit tumor progression in vivo but without drug‐related toxicity. While observing the survival rate, ACEXO significantly improved the survival rate of tumor‐bearing mice (Figure 5C). These in vivo experimental results strongly suggested that ACEXO has a good tumor‐targeting ability, with reduced toxicity and enhanced effect of drugs.

## 4 DISCUSSION

By using aptamer, we addressed a strategy for targeting melanoma CSCs to treat melanoma. Adriamycin is commonly used in treating cancer. However, its toxicity limits its application in clinics. Utilizing exosomes coupled with anti‐CD20 aptamer can significantly enhance the efficacy of Adriamycin with reduced systematic toxicity, which could contribute to the targeted therapeutic strategy.

Though there are size differences for exosomes measured by TEM and DLS methods, the size is within the well‐acknowledged size range. Due to their accessibility and excellent uptake ability, exosomes exhibit great potential in drug delivery,^10^ as shown in this study. The non‐toxicity feature strengthened its usage in the clinic. ACEXO's in vivo accumulation in tumors furthered its potential for targeting therapy due to the use of the aptamer. AEXO showed lower accumulation in tumors, most likely due to the relatively weak ability of AEXO to target and adhere to the tumor tissue. However, AEXO exhibits higher affinity than ACEXO in normal tissues like the liver, spleen, and lung, probably because most ACEXOs had been absorbed by the tumor tissue through the recognition between the aptamer and CD20+ CSCs. However, it might need further investigation into manufacture on a large scale for these engineered exosomes.

Targeted therapy inhibits tumor stem cells by cutting off tumor stem cell conduction pathways, attacking tumor stem cell surface markers, or inducing tumor stem cell differentiation, which opens up new research directions for radical tumor treatment. At present, there are mainly the following targeted treatment pathways, which weaken the self‐renewal ability of CSCs,^11^ destroy the microenvironment of CSCs,^12^ target the surface labeling molecules of CSCs,^13^ enhance the sensitivity of CSCs to radiotherapy and chemotherapy,^14^ and promote the differentiation of CSCs.^15^ Our study aimed to target the surface labeling molecule of CD20+ melanoma tumor stem cells. Nevertheless, this successful technique could also be used for targeting the microenvironment of CSCs.

## 5 CONCLUSION

In this study, exosomes are used as drug delivery carriers, with good biocompatibility, relatively simple preparation, and great application potential. More importantly, in this strategy, CD20 aptamers were used to modify exosomes and enhance exosome specificity. Concluding from our experiments, Adriamycin‐loaded exosomes with anti‐CD20 aptamers exhibit great potential in clinical treatment for melanoma.

## CONFLICT OF INTEREST

The authors declare that they have no conflict of interest.

## Data Availability

The data that support the findings of this study are available on request from the corresponding author.
